# Individual differences in the perception of probability

**DOI:** 10.1371/journal.pcbi.1008871

**Published:** 2021-04-01

**Authors:** Mel W. Khaw, Luminita Stevens, Michael Woodford

**Affiliations:** 1 Center for Cognitive Neuroscience, Duke Institute for Brain Sciences, Duke University, Durham, North Carolina, United States of America; 2 Department of Economics, University of Maryland, College Park, Maryland, United States of America; 3 Department of Economics, Columbia University, New York City, New York, United States of America; Stockholm University, SWEDEN

## Abstract

In recent studies of humans estimating non-stationary probabilities, estimates appear to be unbiased on average, across the full range of probability values to be estimated. This finding is surprising given that experiments measuring probability estimation in other contexts have often identified *conservatism*: individuals tend to overestimate low probability events and underestimate high probability events. In other contexts, *repulsive* biases have also been documented, with individuals producing judgments that tend toward extreme values instead. Using extensive data from a probability estimation task that produces unbiased performance on average, we find substantial biases at the individual level; we document the coexistence of both conservative and repulsive biases in the same experimental context. Individual biases persist despite extensive experience with the task, and are also correlated with other behavioral differences, such as individual variation in response speed and adjustment rates. We conclude that the rich computational demands of our task give rise to a variety of behavioral patterns, and that the apparent unbiasedness of the pooled data is an artifact of the aggregation of heterogeneous biases.

## Author summary

Humans often misrepresent probabilities, frequencies, and proportions they encounter—either overestimating or underestimating the true underlying values. Understanding the cognitive and neural representation of such quantities is important, as probabilities are present in all kinds of impactful decisions—e.g., as we assess the likelihood of a dangerous event or the probable returns on a financial investment. Despite the ubiquitous observation that humans are biased in estimating probabilities, a new laboratory task reportedly elicits unbiased performance. This task involves predicting the likelihood of a green ring emerging from a box containing red and green rings; as subjects draw rings and report their estimates, the ring composition of the box itself changes infrequently. Here we perform a novel and thorough analysis of individuals’ performance on one such experiment, showing that people are indeed biased in idiosyncratic ways. These patterns are unexamined by studies focusing on average performance: some people routinely report an exaggerated range of probabilities, some favor intermediate values, while others are approximately unbiased. These biases persist across hours of experience and accompany other tendencies such as quickness in response. We show that subjects’ idiosyncrasies can be understood as arising from various cognitive mechanisms; nonetheless, subjects’ reports are best described as resembling a distorted version of the optimal statistical estimate.

## Introduction

Decision-makers often report distorted probabilities, despite the ubiquitous day-to-day experience with probability estimation. The type of distortion differs across tasks and contexts, but two commonly identified patterns are *conservatism*, where individuals report probabilities that are less extreme than the true values (tending toward 50%), and *repulsive* biases away from 50%, where extreme values are disproportionately reported. The tendencies of probability estimates to be distorted either around the average value or towards the extremes result in variants of S-shaped response functions.

These seemingly conflicting patterns of biased judgment reflect, at least in part, differences in the tasks analyzed. For example, the conversion of experienced frequencies into reported probabilities tends to be associated with conservatism [1], while the conversion of numerical proportions into visual representations results in the opposite pattern from that of judgment errors [2]. In general, studies involving the estimation of an observed frequency often find conservatism, such as in visual judgments of dots of different colors [3, 4], sequences of letters [5], and ratios of auditory durations [6]. Reversals of this bias have also been documented, though they are less common [2, 7, 8]. In the probability calibration literature, overconfidence has been documented more frequently (for a review, see [9]). These experiments involve asking subjects a question about a unique event (e.g., a general knowledge question). Subjects are also asked to assign a probability that their answer is correct (e.g., [10]). Overconfidence is indicated when confidence ratings (e.g., how likely the subject thought their answer was correct) are greater than the frequency of correct answers. Also in this domain, the modal bias can reverse, depending on the difficulty level of questions [11].

The inferred bias also depends on the statistical approach used: Both repulsive errors and conservatism can be identified within the same data set, depending on the statistics used to characterize bias [12]. In addition, judgments are subject to internal noise, which can interfere with the identification of biases [12–14].

Further complicating matters, recent research on the estimation of non-stationary probabilities has emphasized how nearly unbiased estimates are on average—while recognizing that estimates on individual trials are noisy [15–17]. In these studies, aggregating probability estimates across subjects yields a median forecast that seems to closely track the underlying true probability, over the full range of possible probability values.

In this paper, we probe the surprising finding of unbiased forecasting of non-stationary probabilities by studying the subject-level data from Khaw et al. [17], who present results from an estimation task similar to that of Gallistel et al. [15]. The experimental design includes elements of probability estimation as well as sequential change detection [18]. Subjects observe many realizations of a Bernoulli random variable, in the form of draws of red or green rings from a virtual box. They are asked to indicate the probability of drawing a green ring on each draw. The true probability itself changes from time to time in an unsignaled manner. Subjects achieve relatively unbiased average performance relative to both the true probabilities and the Bayesian benchmarks [15–17]. This is surprising given the added computational complexity of the task compared to those used in prior studies, discussed above.

The main questions studied in this paper are the following: Is probability estimation unbiased when examined at the individual level? If this is not the case, what are the ways in which individual estimates deviate from the unbiased forecast in this paradigm? The large number of observations for each subject allows us to analyze these data at the subject level, testing for a variety of individual biases. Additionally, we are able to test if differences are stable within individuals, despite extensive experience with the task, and whether they are correlated with other individual patterns of behavior. We also test whether observed biases might be accounted for by alternative theories of probability estimation; we consider two major classes of models, centered on either error-based updating or limited samples of prior observations.

We characterize the data using a family of computational models of subjective probability estimation, based on the probabilistic log odds model [12, 19]. The models accommodate random noise in subjective estimates, as proposed by a number of existing models in the literature [12, 13, 20, 21]. Importantly, we allow for a general form of conservatism or repulsion, a flexible cross-over point between over-weighing and under-weighting (or “indifference point”, as defined by Attneave [1]), and for a wide range of heterogeneity across subjects. This flexibility allows us to identify the most important dimensions along which subjects’ estimation performance deviates from unbiased estimates. Our approach is based on work that has proposed non-linear probability weighting functions to account for biases in the treatment of probability. These models specify subjective decision weights as implied by choice behavior [22–24], or describe how probability is estimated from perceived frequencies.

We find that unbiasedness is an artifact of aggregating the behavior of a balanced sample of individually biased subjects. The estimates of individual subjects are often far from unbiased, but the biases of different subjects are quite different, in a way that can allow the pooled data to look nearly unbiased. We conclude with an analysis that further links the types of biases identified to other metrics of behavior in the task. Our results regarding the coexistence of a range of biases across subjects completing the same task complement those of Zhang, Ren, and Maloney [25], who find that the type of bias may change across tasks for the same participant. Varying levels of individual accuracy have been reported in the same kind of task by Forsgren, Juslin, and Van Den Berg [26] but not analyzed in detail in this respect.

## Methods

### Data description

We analyze the individual-level data from Khaw et al. [17] made publicly available through the associated *Data In Brief* supplement. The task is a modified version of Gallistel et al.’s [15] experimental design. Subjects were instructed to estimate the probability of drawing a green ring out of a box with green and red rings. The Bernoulli parameter governing this probability changed occasionally, in an unsignaled manner, over the course of each session. After each ring draw, there was a fixed probability of 0.05 that a new box would be created, with a new proportion of green rings. This non-stationarity required subjects to consider what the evidence presented implied for both their current forecast and for the possibility that they are drawing from an entirely different box. The subjects were told about this rate of change, and that each time there was a change, new values for the true probability would be drawn from the uniform distribution on the unit interval.

Subjects’ estimates of these probabilities and their response times were recorded for each ring draw, and subjects were given monetary rewards for the accuracy of their estimates. Notably, unlike other trial-by-trial experimental procedures, the task was self-paced: subjects clicked a button (labeled ‘NEXT’) to request the next ring draw. The reported probabilities were measured using each subject’s slider position at the time the ‘NEXT’ button was clicked; response times denote the time elapsed between clicks requesting the next ring. We have 10,000 observations for each subject, which gives us enough data to conclude with some confidence that any systematic patterns we identify are not the result of random noise. The data set contains estimates reported by 11 subjects, each of whom completed 10 sessions of 1,000 draws each. Khaw, Stevens, and Woodford [27] report additional details about the experimental paradigm, including figures of the user interface.

**Fig 1:**
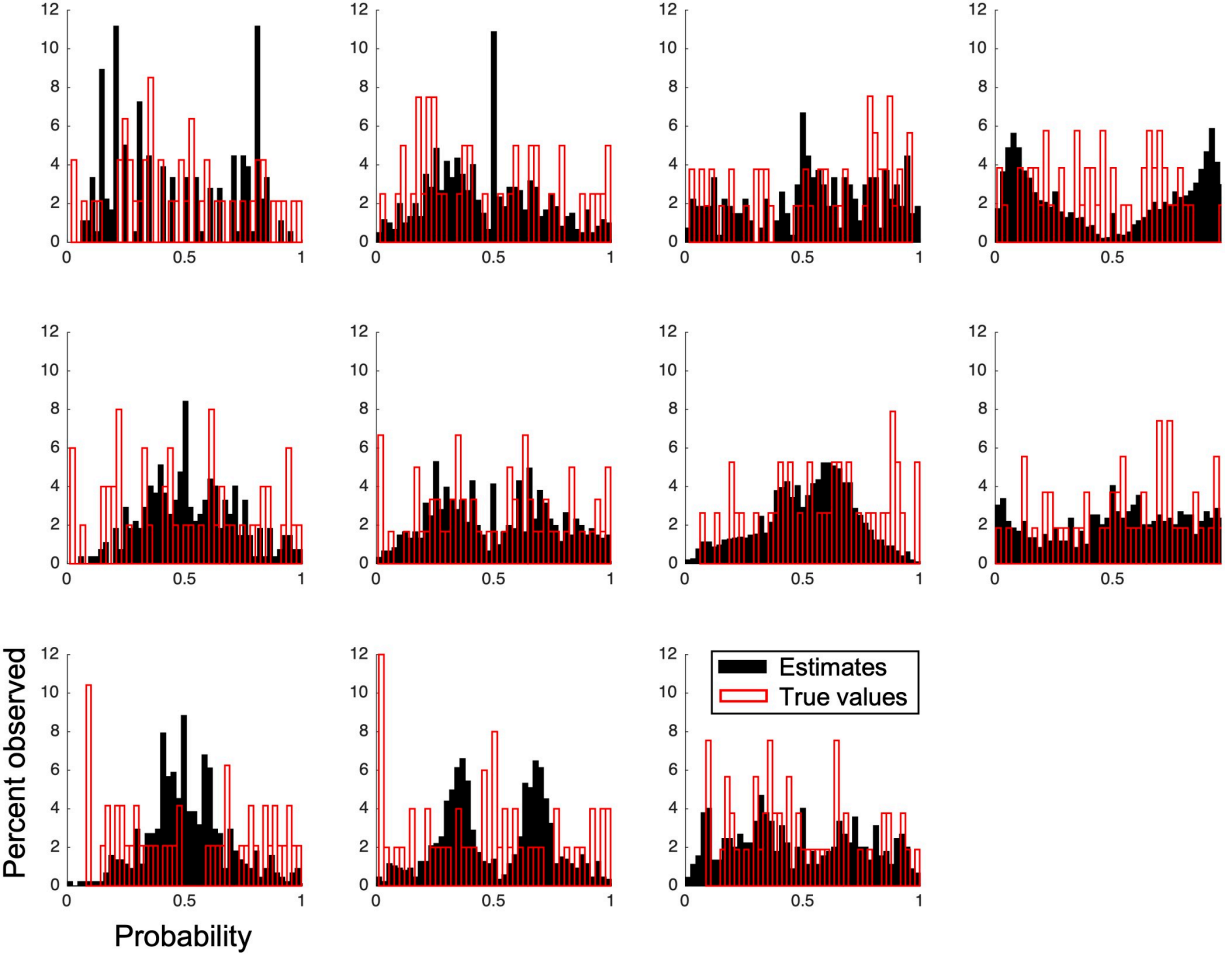
The subject-level distributions of reported probabilities and the ‘true’ underlying probabilities in the dataset of Khaw, Stevens, and Woodford [27]. In contrast to the distributions of probabilities reported by individual subjects, the underlying probabilities were drawn uniformly from the unit interval. Deviations from the uniform distribution in the underlying probabilities reflect finite sampling.

Fig 1 shows the distributions of probability estimates for individual subjects. Individuals show a wide range of estimates over the course of the task. Some subjects, such as subjects 5 and 9, tend to concentrate around 0.5, while others, such as subjects 4 or 10, avoid 0.5. In contrast to these subjective distributions, the true probabilities of drawing a green ring were drawn uniformly from the unit interval.

**Fig 2:**
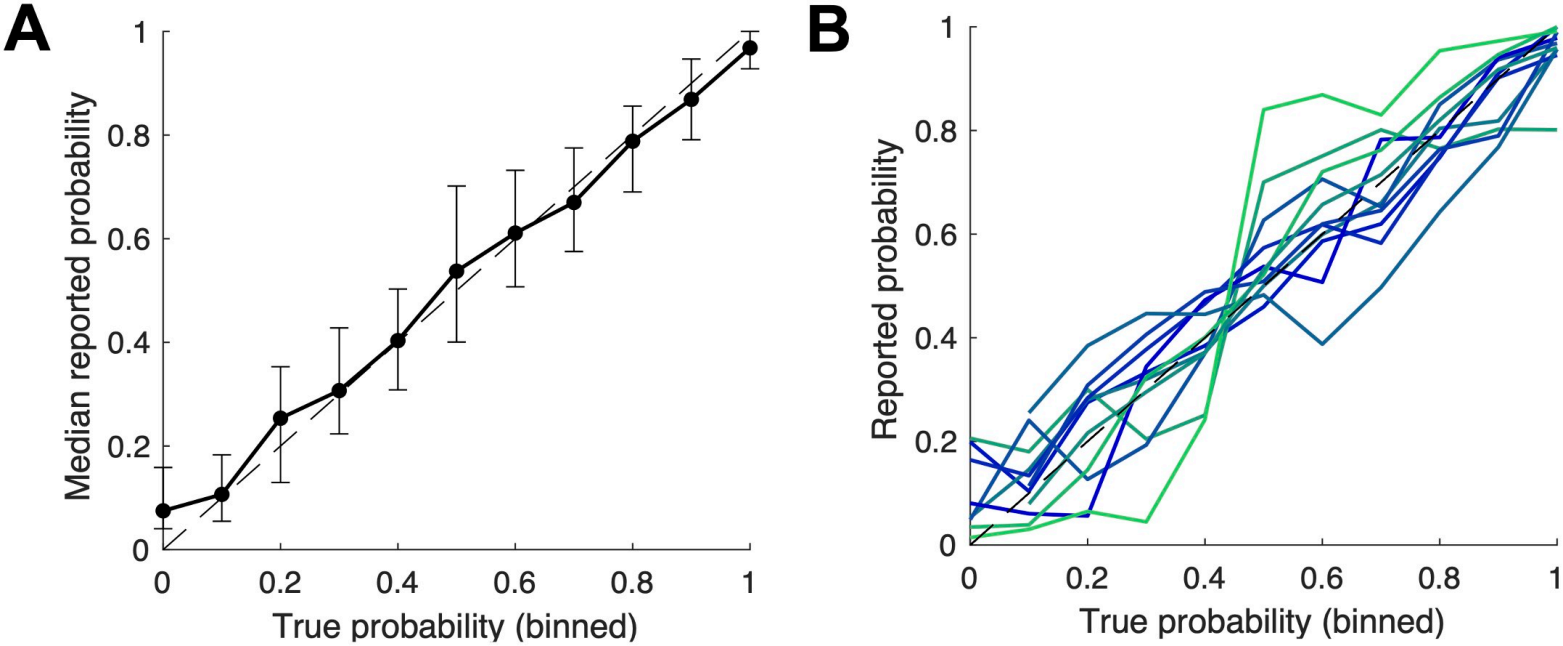
Pooled and individual-level probability estimates. (A) The median estimate across the pooled sample tracks the true probability in each bin. The error bars denote interquartile ranges. (B) The median response of each participant, across trials and sessions, generates varying S-shaped response functions. Participants are color-coded by their degree of conservatism, with darker colors indicating more conservatism (as defined in the *Computational Models* section).

To visualize the overall level of accuracy achieved on the task, Fig 2A plots the median reported probability against the true underlying probability. The proximity to the 45 degree line supports the observation of Gallistel et al. [15] that the mapping between true values and perceived probability is approximately “the identity function over the full range of probabilities.” However, plotting the median forecast at the participant level (aggregating across trials and sessions) gives rise to a wide range of biases in the form of varying S-shaped distortions around the 45 degree line (Fig 2B). It is important to emphasize that the individual mappings are constructed based on 10,000 trials for each subject, and thus do not represent inherent noise across trials, which would wash out over such a large sample. These differences foreshadow the more formal results of both conservatism and repulsive biases, discussed in the next section.

### The forecasts of a Bayesian observer

The participants cannot directly observe the true probability of drawing a green ring on each trial. Instead, they are given information about the ring generating process and observe a sequence of random ring draws. The ring draws are noisy signals that generate a degree of randomness relative to the true probability even for an ideal observer. Moreover, the presence of this randomness may itself induce participants to optimally distort their forecasts relative to the true probability, just as a Bayesian statistician puts a lower weight on a noisier signal. We are interested in how well participants perform given what they observe, how well they use the information available to them. Hence, we focus our analysis not on how closely participants forecast the true probability, but rather on how close their forecasts are to those of an ideal observer, who computes probabilities optimally using all the available information.

We consider an ideal observer who is given exactly the same information as our participants, and who forms optimal Bayesian forecasts, based on (*i*) knowledge of the process that generates the rings and (*ii*) the history of ring realizations observed. Relevant to these forecasts are both the fact that the true probability is drawn uniformly from the unit interval, and the fact after each draw there is a 0.5% probability that the true probability is replaced by an independent draw, also from the unit interval. The Bayesian forecast starts at 0.5 and is updated after each ring realization. The solution to the Bayesian benchmark is derived in Khaw et al. [17] and reproduced in S1 Appendix.

**Fig 3:**
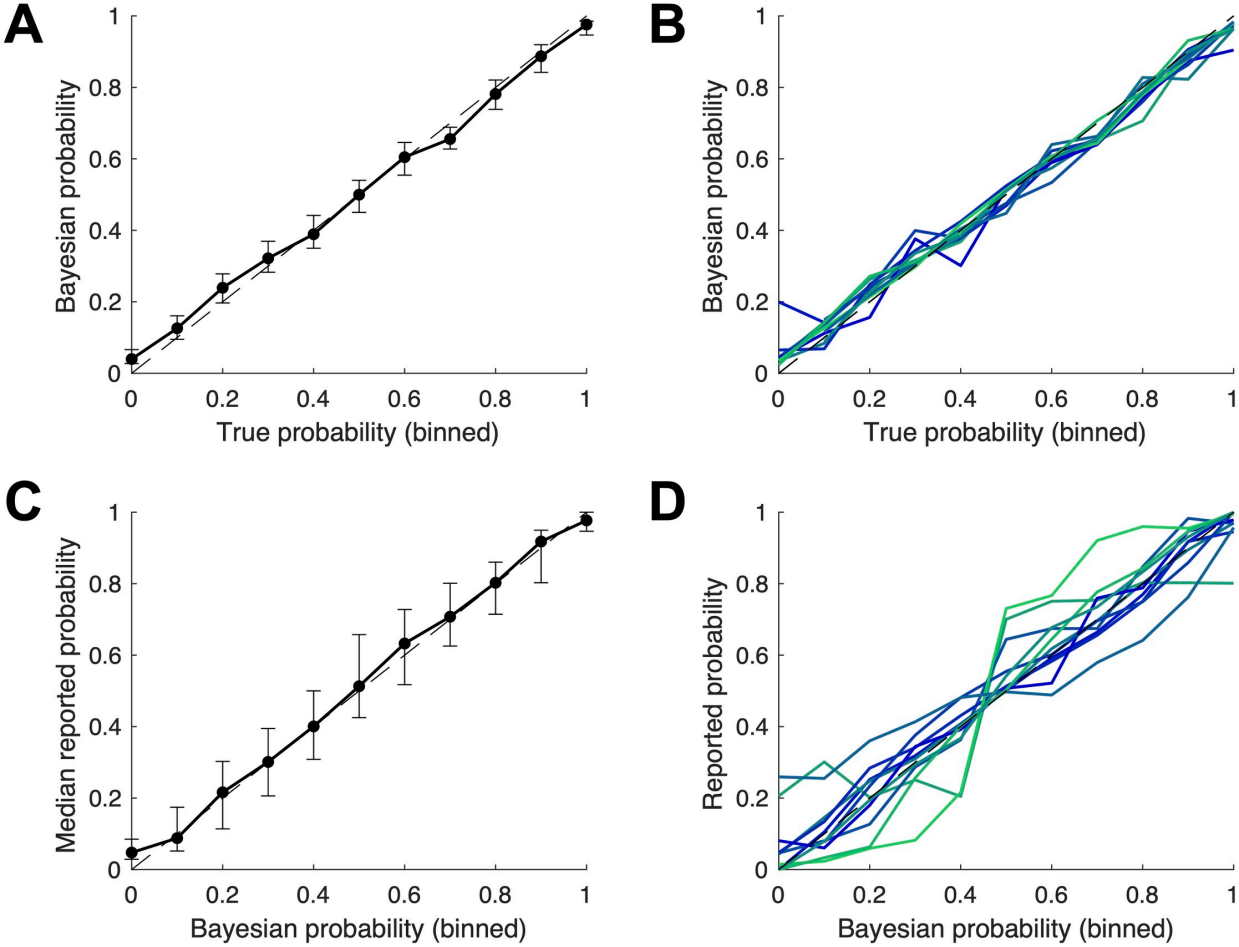
The Bayesian forecast and true probabilities. (A) The median Bayesian estimate closely tracks the true underlying probability in each bin. (B) The median Bayesian responses corresponding to the sessions completed by each individual generate very small dispersion around the diagonal. (C) and (D) Much of the deviation in the subjective probability forecasts from the true probabilities reflect deviations from the Bayesian forecast.

The randomness introduced by the ring realizations generates a slight dampening in the Bayesian forecasts, as shown in the top panels of Fig 3. However, the deviations of the Bayesian forecast from the true probability are much smaller than those of our participants. As demonstrated by the lower panels of Fig 3, most of the divergence between subjective forecasts and the true probability reflects deviations of the subjective forecasts from the optimal forecast, *not* deviations of the optimal forecast from the true probability.

### Computational models

In order to formally test for and characterize potential biases in these data, we turn to a model comparison exercise. We consider a family of computational models that describe the subjective probability estimates reported by our subjects as a noisy function of the ideal Bayesian observer’s estimate. Many authors have proposed that psychological probabilities can be proxied by nonlinear transformations of the objective probability [22–24, 28]. We use the linear log odds representation of probabilities, which is consistent with a wide range of data on subjective probability estimates [12, 19, 25].

Letting a subject’s reported probability be denoted by *R*, we estimate the model

$$\begin{array}{c} log(\frac{R}{1-R})=\alpha +\beta \times log(\frac{B}{1-B})+\varepsilon , \end{array}$$ (1)

where *B* is the Bayesian estimate, and the random noise in subjects’ estimates is assumed to be normally distributed with zero mean and variance *σ*^2^ across trials, $\varepsilon ∼N(0,\sigma^{2})$.

**Fig 4:**
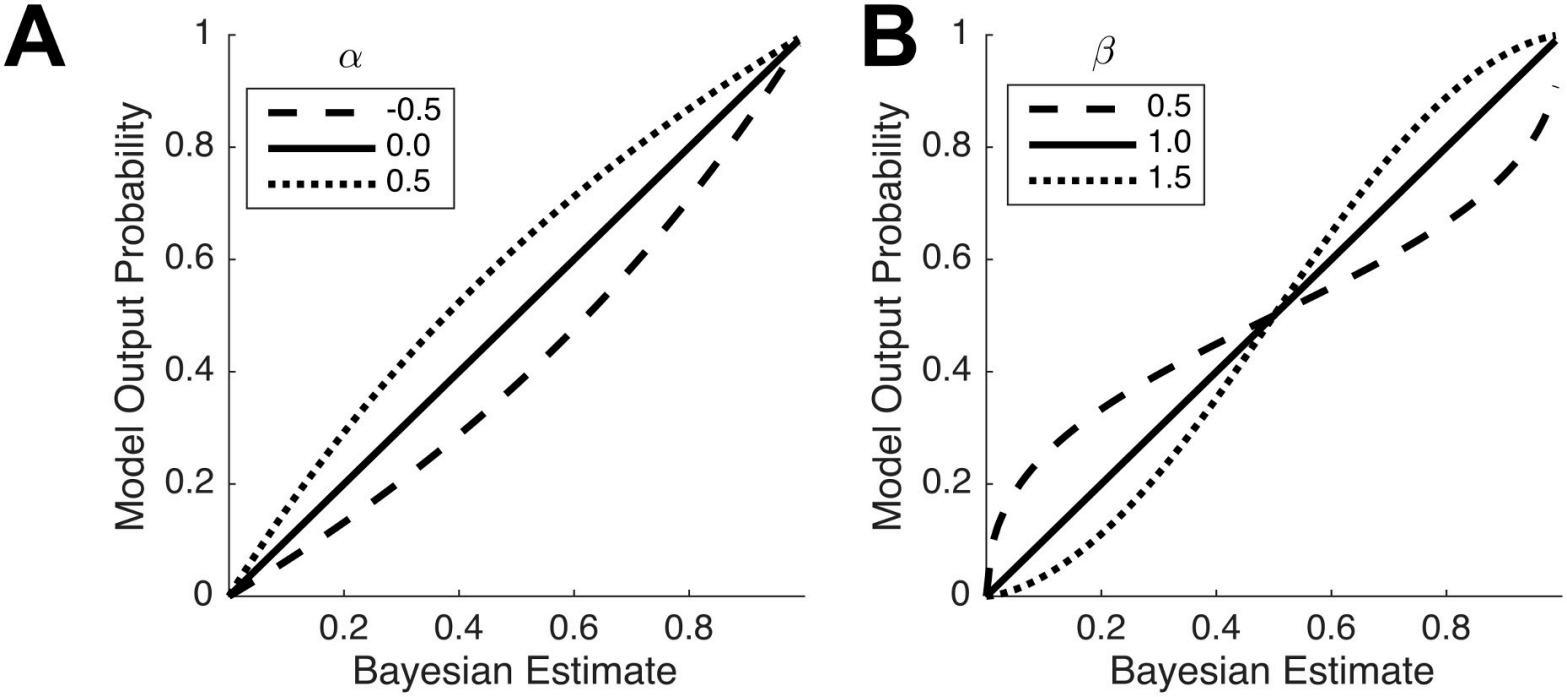
The two types of estimation biases accommodated by the free parameters of the tested models. (A) The additive bias implied by non-zero values of *α*. (B) The non-linear distortion toward or away from 0.5, implied by *β* different from 1.

We allow for two types of systematic distortions. First, the parameter *α* allows for uniform over- or underestimation: non-zero values of *α* predict that a subject’s estimates will systematically be either higher or lower than the Bayesian response (Fig 4A). Second, the parameter *β* governs the extent to which estimates favor the center of the scale, characteristic of conservatism (*β* < 1), or extreme values, which would indicate a repulsive bias (*β* > 1; Fig 4B). This specification is similar to that used by Offerman and Sonnemans [20], who analyze subjects’ estimates following observations of coin flip outcome sequences.

Individual subjects adjust their forecast infrequently, as documented by Khaw, Stevens, and Woodford [17]. The unconditional frequency of adjustment is 8.8 percent, despite the steady stream of new ring realizations and the fact that the Bayesian forecasts adjust, however modestly, in response to each new ring draw. Infrequent adjustment often arises in experiments that feature long series of repeated trials [16, 26]. While not the focus of our study, it needs to be taken into account, to avoid mischaracterizing subjective biases. We estimate Eq (1) both unconditionally, on the full series, and conditional on adjustment. The conditional estimation uses the subjective and Bayesian forecasts recorded at the time of the subjective adjustment, and discards forecasts between adjustments.

We consider variants of Eq (1) that accommodate different combinations of biases as well as different degrees of heterogeneity across the key parameters *α*, *β*, and *σ*.

#### Homogeneous models

We first estimate a set of models that impose common parameter values across the entire participant sample. We begin with the unbiased specification, in which we estimate *σ* ≠ 0 but impose *α* = 0 and *β* = 1. Next, we allow one or both bias parameters to deviate from their null values, and we estimate the values that best fit the pooled data.

#### Heterogeneous models

The next set of specifications allow parameters to differ across subjects. We first allow the level of noise *σ* to vary across subjects, while imposing homogeneity in *α* and *β*. Next, we re-estimate the models allowing for bias heterogeneity across different parameter sets. Finally, we estimate individual values {*α*_*i*_, *β*_*i*_, *σ*_*i*_}, for each subject *i*. Our goal in this analysis is to determine which dimension of heterogeneity matters most in characterizing individual adjustment patterns.

In addition, results are compared to the *Random* benchmark, showing the likelihood of the observed responses, if responses were drawn randomly (thus imposing *α* = 0 and *β* = 0). The estimated parameters maximize the log likelihood of observing the data given the functional specification of each model. The models are ranked using the Bayes Information Criterion (BIC), to penalize model complexity. Since we use a log odds transformation, we code all reports of certainty to be either 0.99 or 0.01.

## Results

### Model estimation results

The models’ free and fixed parameters, along with the log-likelihoods and BIC values are reported in Table 1. For the heterogeneous parameters models, the table reports the average value across subjects. The top panels of the table report unconditional results, while the bottom panels report estimation results conditional on subjects adjusting their forecasts. Subjective forecasts exhibit considerable stochasticity: responses to identical realizations of the Bernoulli variable differ, even for the same subject. Because of this randomness in forecasting, we omit reporting results for models that impose *σ* = 0. We highlight five key findings.

**Table 1:** Model estimates.

Model	*α*	*β*	*σ*	*k*	−*LL*	*BIC*
Unconditional Estimates						
Homogeneous Models						
Random benchmark	0	0	1.896	1	226024	452060
Estimated *σ*	0	1	0.947	1	149812	299635
Estimated *α*, *σ*	0.028	1	0.947	2	149762	299548
Estimated *β*, *σ*	0	0.987	0.947	2	149782	299588
Estimated *α*, *β*, *σ*	0.031	0.985	0.946	3	149723	299481
Heterogeneous Models						
Estimated {*α*_*i*_}, *β*, *σ*	**0.031**	0.984	0.942	13	149192	298536
Estimated *α*, {*β*_*i*_}, *σ*	0.040	**0.997**	0.831	13	135496	271144
Estimated *α*, *β*, {*σ*_*i*_}	0.053	0.949	**0.897**	13	137560	275271
Estimated {*α*_*i*_}, *β*, {*σ*_*i*_}	**0.038**	0.947	**0.893**	23	137174	274616
Estimated *α*, {*β*_*i*_}, {*σ*_*i*_}	0.055	**0.996**	**0.798**	23	126438	253143
Estimated {*α*_*i*_}, {*β*_*i*_}, {*σ*_*i*_}	**0.039**	**0.999**	**0.796**	33	126192	252767
Conditional Estimates						
Homogeneous Models						
Random benchmark	0	0	1.597	1	18253	36515
Estimated *σ*	0	1	1.039	1	14091	28191
Estimated *α*, *σ*	0.092	1	1.035	2	14053	28125
Estimated *β*, *σ*	0	1.152	1.026	2	13973	27964
Estimated *α*, *β*, *σ*	0.098	1.155	1.021	3	13929	27885
Heterogeneous Models						
Estimated {*α*_*i*_}, *β*, *σ*	**0.078**	1.162	1.018	13	13893	27906
Estimated *α*, {*β*_*i*_}, *σ*	0.128	**1.019**	0.912	13	12836	25792
Estimated *α*, *β*, {*σ*_*i*_}	0.066	0.950	**0.926**	13	12797	25714
Estimated {*α*_*i*_}, *β*, {*σ*_*i*_}	**0.078**	0.952	**0.920**	23	12772	25755
Estimated *α*, {*β*_*i*_}, {*σ*_*i*_}	0.083	**1.018**	**0.839**	23	11773	23758
Estimated {*α*_*i*_}, {*β*_*i*_}, {*σ*_*i*_}	**0.090**	**1.026**	**0.832**	33	11707	23718

Note: The parameter values fixed to null values are shown in gray. For estimations that allow parameters to differ across subjects, the table reports the averages across subjects, in bold. *LL* is the log-likelihood, *k* is the number of free parameters, and *BIC* = −2*LL* + *kln*(*N*). The number of observations is *N* = 109, 780 for the unconditional estimates and *N* = 9, 673 for estimates conditional on adjustment.

First, the representation of subjective estimates as noisy and biased functions of the optimal Bayesian forecast represents the data well in our sample. The subjective forecasts track the Bayesian optimal estimate (albeit noisily), substantially outperforming the random forecast benchmark. Moreover, the models that allow for both additive and nonlinear biases yield a better fit, even with a model-complexity penalty, compared with the model that imposes no systematic biases and only allows for random noise around the Bayesian forecast.

Second, we estimate substantial positive additive bias and repulsion, once we take into account the infrequent adjustment of subjective estimates. The homogeneous model estimates are *α* = 0.098 and *β* = 1.155 when estimated conditioning on adjustment, compared with *α* = 0.031 and *β* = 0.985 for the unconditional sample. These differences reflect the infrequent adjustment in these subjects’ forecasting decisions, which dampens the estimated sensitivity of the subjective forecast to the objective probability. We focus our estimation on the sample of subjective probabilities conditional on adjustment, so as not to confound infrequent adjustment with conservative adjustment.

Third, bias heterogeneity is a significant feature of these data. Models that allow for individual-specific parameters outperform those that impose common values across subjects. In fact, the model that allows for individual-specific values for all three parameters explains the data best among those tested here, once again, even after penalizing for model complexity. Cross-sectional variations in the non-linear bias parameter *β* and in the standard deviation of noise *σ* generate the biggest improvements in model fit.

Fourth, there is an interaction between the parameters: imposing homogeneity in the bias parameters results in higher estimates of the level of noise. Gallistel et al. [15] also implicitly point out the connection between non-linear bias and noise. As they note, the more sensitive subjective estimates are to the data (which, in our framework, translates into a higher value for *β*), the noisier will be the individual forecasts, especially in this binary outcome setting.

Fifth, accounting for heterogeneity recovers the relatively unbiased average performance in this sample: in the model with heterogeneity in all three model parameters, we estimate only modest degrees of positive bias *α* = 0.09 and repulsion *β* = 1.026, on average. In the later section on individual-level biases, we document the degree of variability around these average values.

### Comparison to second-best specifications

We can furthermore consider the merit of full heterogeneity (across all three bias parameters) with a four-fold cross-validation exercise. The key comparison here involves the full model and the second-best model—a variant that assumes homogeneity in the additive bias parameter *α*.

We consider the in-sample fit of these models by selecting a subset comprising three fourths of randomly chosen observations (the “calibration” dataset), and then finding the parameter estimates that maximize the likelihood of these observations. We evaluate the out-of-sample fit of these models by computing the likelihood of observing the data in the remaining one fourths of each dataset (the “validation” dataset), using the fitted calibration parameters. In Table 2, the columns titled *BIC*^*calibration*^ and *BIC*^*validation*^ respectively report the average *BIC* values obtained from the calibration datasets and the remaining validation partitions. We follow Khaw, Li, and Woodford [29] in reporting a composite Bayes factor *K* = *K*_1_ ⋅ *K*_2_, taking into account observations of both calibration and validation datasets. For any two models, $M_{1}$ and $M_{2}$

$$\begin{array}{c} \log K_{1}=LL^{calibration}\left(M_{2}\right)-LL^{calibration}\left(M_{1}\right)-\left(k_{1}-k_{2}\right)\ln\left(N^{calibration}\right) \end{array}$$ (2)

$$\begin{array}{c} \log K_{2}=LL^{Validation}\left(M_{1}\right)-LL^{Validation}\left(M_{2}\right) \end{array}$$ (3)

**Table 2:** In-sample and out-of-sample measures of goodness-of-fit compared for the fully heterogeneous model and close alternatives.

Model	*BIC*^*calibration*^	*BIC*^*validation*^	Log K
Estimated {*α*_*i*_}, {*β*_*i*_}, {*σ*_*i*_}	17749	6063	0
Estimated *α*, {*β*_*i*_}, {*σ*_*i*_}	17857	6101	67
Estimated {*β*_*i*_}, {*σ*_*i*_}	17877	6065	93
Estimated {*α*_*i*_}, *β*, {*σ*_*i*_}	19358	6593	1064

In this formulation, $M_{1}$ is the model allowing for full heterogeneity, while $M_{2}$ is the alternative model considered on each line of Table 2; values *K* > 1 indicate the degree to which the data provide more support for the fully heterogeneous model than for the alternative. In (2), *k*_1_ and *k*_2_ are the numbers of free parameters in the respective models and *N*^*calibration*^ is the number of observations in each calibration dataset (in effect, penalizing for a greater number of free parameters following the *BIC* formula). The logarithm of the composite Bayes factor K is reported in the final column of Table 2, as an overall summary of the degree to which the data provide support for each model.

We find that for both in and out-of-sample fits, the fully heterogeneous model outperforms competitive variants that do not allow for heterogeneity in the additive bias parameter. The Bayes factor *K* thus also offers a relative likelihood that summarily supports the full model.

### Individual biases in probability judgments

**Fig 5:**
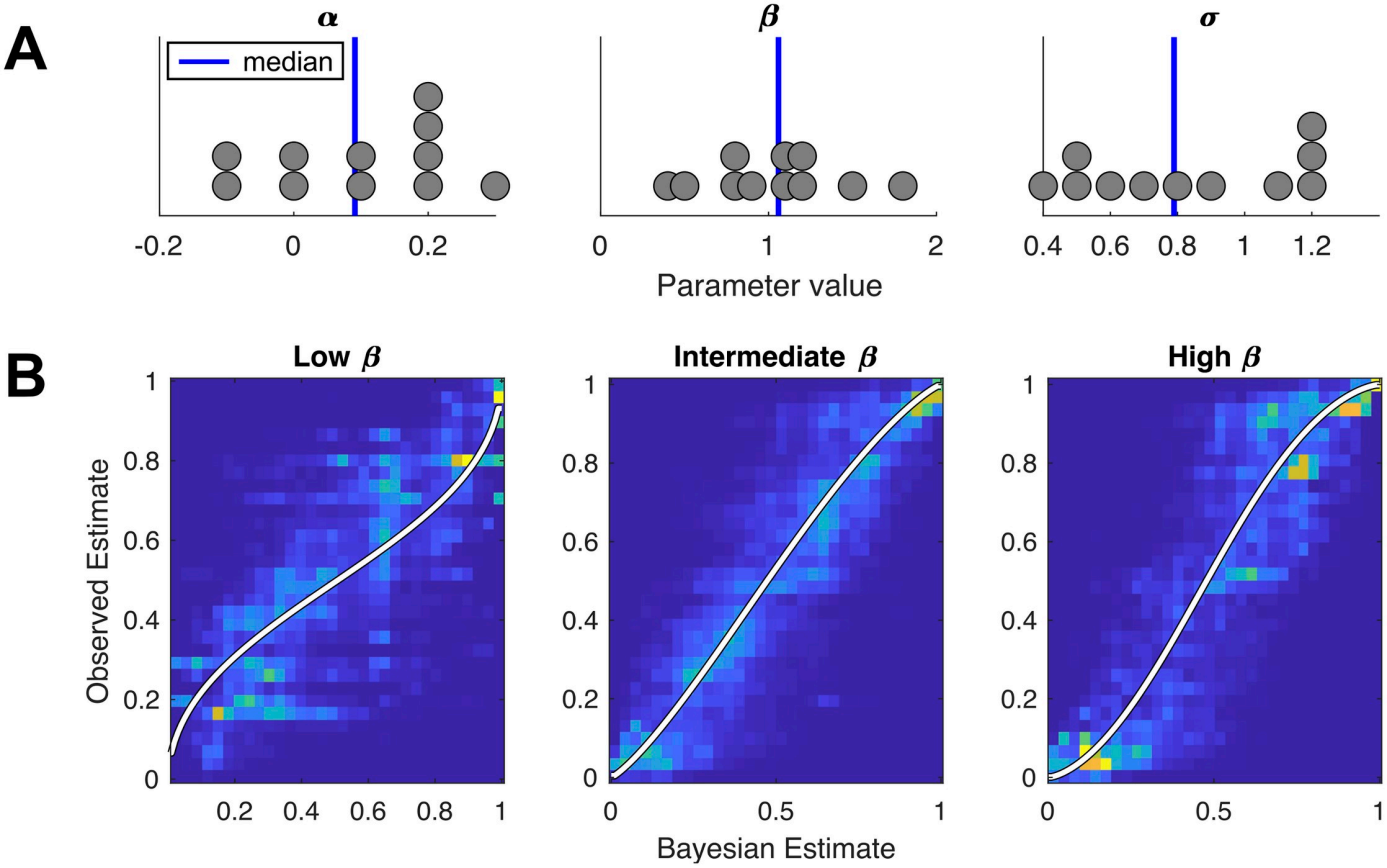
Bias parameter distributions and subjects’ response densities. (A) Dot plots showing the distributions of best-fitting parameters (*α*, *β*, *σ*) across subjects. (B) The density of responses corresponding to members between terciles of *β* values. Bold colored lines indicate the output produced by the model in Eq (1) using the median model parameters from each tercile.

We now turn to individual biases, focusing on results estimated conditional on adjustment. Fig 5A plots the distribution of parameter values for the best-fitting model. Consistent with a large body of work that has documented dispersion and stochasticity in decision-making, we find considerable noise in subjects’ individual estimates relative to the Bayesian model. The standard deviation of errors ranges between 0.44 and 1.21. More importantly, we identify systematic biases that do not seem to reflect random noise in the execution of the experimental task. We estimate nonzero values for the intercept bias *α* ranging from 0.02 to 0.27 in absolute value. For all but two participants, *α* = 0 lies outside the 95% confidence interval. One participant has a statistically significant negative additive bias, while the remainder tend to report forecasts that are systematically higher than the Bayesian forecast.

The non-linear distortion parameter *β* ranges from 0.41 to 1.82 across participants, despite extensive experience with the task, over the course of 10,000 trials. For one participant, the no-bias value (*β* = 1) lies inside the 95% confidence interval, while the remaining participants are evenly split between statistically significant conservatism and repulsion. Fig 5B plots the distribution of individual responses against the Bayesian estimates, for each tercile of the distribution of *β* values. The conservative values in our sample are comparable to the values of 0.56, 0.61 and 0.71 reported by Gonzalez and Wu [23] in prior experiments, while the repulsive values suggest an excessive sensitivity to ring realizations over the course of the trials.

The strong biases at the individual level end up canceling out in the group average. However, this group average is based on a relatively small number of participants. While we have strong statistical evidence in support of biases at the individual level, given the large number of trials per subject, we cannot say with any certainty that the canceling out of biases across participants would survive in another sample of participants. The fact that biases are so correlated across trials for each participant substantially reduces the number of independent observations that can be claimed when making statements about the group averages.

We report subject-level parameter estimates in Table 3. The table also reports bootstrapped confidence intervals around each subject’s bias parameters. The parameters were re-estimated for 1, 000 iterations, using data that were sampled with replacement from each subject’s original data set. The relevance of individual biases does not diminish by either (*i*) estimating confidence intervals by averaging over session-wise data (reported in the last columns of the table) or (*ii*) computing credible regions that are within 2 log likelihood points from the best-fitting set of parameters. In terms of individual model fits, all subjects are best fit by the model that allows for full heterogeneity—S1 Table contains model fit statistics and second-best models at the subject level.

**Table 3:** Individual parameter estimates.

Subject	Parameter	M.L. Estimate	95% C.I.	Mean Estimate	S.E.
1	*α*	0.091	[0.031, 0.152]	0.193	0.087
	*β*	1.059	[0.994, 1.131]	1.154	0.120
	*σ*	0.790	[0.707, 0.876]	0.691	0.062
2	*α*	-0.130	[-0.286, -0.011]	0.057	0.166
	*β*	0.407	[0.275, 0.542]	0.758	0.314
	*σ*	1.208	[1.131, 1.276]	1.037	0.128
3	*α*	0.044	[0.011, 0.074]	0.053	0.029
	*β*	0.905	[0.875, 0.933]	0.904	0.029
	*σ*	0.443	[0.414, 0.471]	0.398	0.022
4	*α*	0.198	[0.135, 0.267]	0.065	0.118
	*β*	1.124	[1.032, 1.221]	1.389	0.156
	*σ*	1.088	[1.006, 1.163]	1.014	0.047
5	*α*	0.159	[0.090, 0.218]	0.107	0.074
	*β*	0.814	[0.724, 0.907]	0.897	0.067
	*σ*	0.548	[0.469, 0.632]	0.454	0.055
6	*α*	0.196	[0.069, 0.325]	0.223	0.084
	*β*	1.203	[1.099, 1.307]	1.288	0.137
	*σ*	1.161	[1.042, 1.280]	1.081	0.098
7	*α*	0.050	[0.025, 0.075]	0.158	0.060
	*β*	0.810	[0.775, 0.842]	0.776	0.086
	*σ*	0.576	[0.549, 0.601]	0.477	0.029
8	*α*	0.267	[0.221, 0.314]	0.342	0.158
	*β*	1.823	[1.771, 1.880]	2.026	0.199
	*σ*	1.167	[1.131, 1.200]	0.966	0.076
9	*α*	0.018	[-0.023, 0.060]	-0.047	0.150
	*β*	0.498	[0.445, 0.550]	0.366	0.068
	*σ*	0.517	[0.459, 0.566]	0.363	0.055
10	*α*	-0.059	[-0.130, 0.017]	0.135	0.191
	*β*	1.460	[1.392, 1.520]	1.456	0.154
	*σ*	0.930	[0.869, 0.989]	0.854	0.041
11	*α*	0.161	[0.092, 0.227]	0.189	0.070
	*β*	1.186	[1.132, 1.245]	1.193	0.146
	*σ*	0.727	[0.643, 0.806]	0.602	0.058

Note: Individual estimates and bootstrapped 95 percent confidence intervals. Means and standard errors are computed from session-wide maximum likelihood estimates.

In light of the large biases estimated at the individual level, it is all the more surprising that the average sample behavior is unbiased. Our results are consistent with the theory of rational expectations proposed by Muth [30], according to which aggregated estimates are close to the normative Bayesian benchmark, with individual biases being distributed in a way that “washes out” in the pooled analyses. We confirm this using the Wilcoxon Signed Rank Test, a non-parametric difference test between fitted parameter values against their null benchmark values. Inspecting the distributions of individual-specific parameters implied by responses unconditional on adjustment (as seen in Gallistel et al.’s [15] aggregation and our replication in Fig 2A), the average value of *α* does not differ significantly from zero (*Z* = 53, *p* = 0.083), and likewise, the average value of *β* does not differ significantly from the benchmark value of *β* = 1 (*Z* = 34, *p* = 0.97).

### Bias stability across sessions

We next address the issue of how stable biases are across sessions completed by the same individual. We begin by testing if the session-level best-fitting parameters are as variable within subjects as they are across subjects. If that were the case, then the heterogeneity captured at the subject-level would reflect random variation at the session level rather than systematic patterns of (subject level) behavior. To investigate this, we exploit the large quantity of data available at the subject level. Each subject performed 10 sessions of the task and submitted 1,000 forecasts for each session. We fit the full model to each individual session of data to yield 110 parameter triplets.

**Fig 6:**
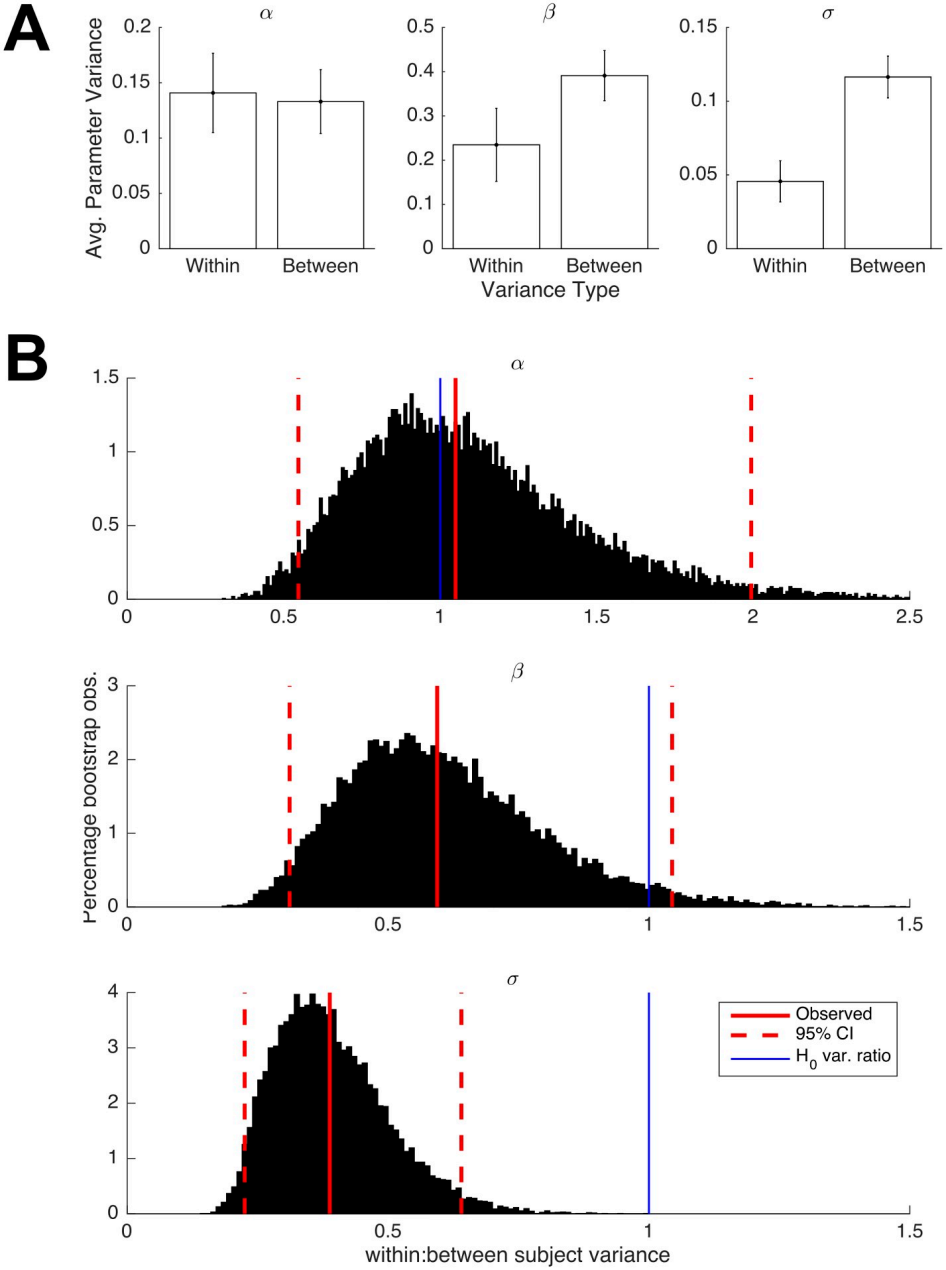
Examination of within and between-subject variability in session-wise parameter estimates. (A) Average between-subject variance is greater than within-subject variance by a factor of around two for parameters *β* and *σ*, with the latter difference being statistically significant. (B) Non-parametric bootstrap tests mirror the observed differences in variance ratios from the null benchmark.

Testing whether average levels of within-subject variability were greater than between-subject variability, we find no significant difference for values associated with the additive bias parameter (*t*(19) = 0.17, p = 0.57). We find partial support for within-subject variability being smaller for the *β* parameter (*t*(19) = -1.53, p = 0.07), noting the non-statistically significant p-value. This difference is significant for the variance associated with the noise parameter *σ* (*t*((19) = -3.55, p < 0.01). The average between-subject variance for these two parameters were approximately twice as large as the within-subject variance (Fig 6A). Bootstrap tests—sampling parameters with replacement from both within and between-subject partitions of each distribution of parameters—corroborate the observed differences in variability (Fig 6B shows distributions of variance ratios from 10, 000 bootstrap iterations). Mirroring the previous results of observed differences, bootstrapped ratios of within to between-subject variability were not significantly different for the *α* parameter (*F*_*boot*_ = 1.05, 95%*CI* = [0.55, 1.98]); we find a difference that was close to significance for *β* (*F*_*boot*_ = 0.59, 95%*CI* = [0.31, 1.05]) and a significant difference for *σ* (*F*_*boot*_ = 0.38, 95%*CI* = [0.22, 0.64]). Taken together, we find evidence for a smaller degree of within-subject variability for the noise parameter, with a similar difference close to statistical significance for the parameter *β*.

**Fig 7:**
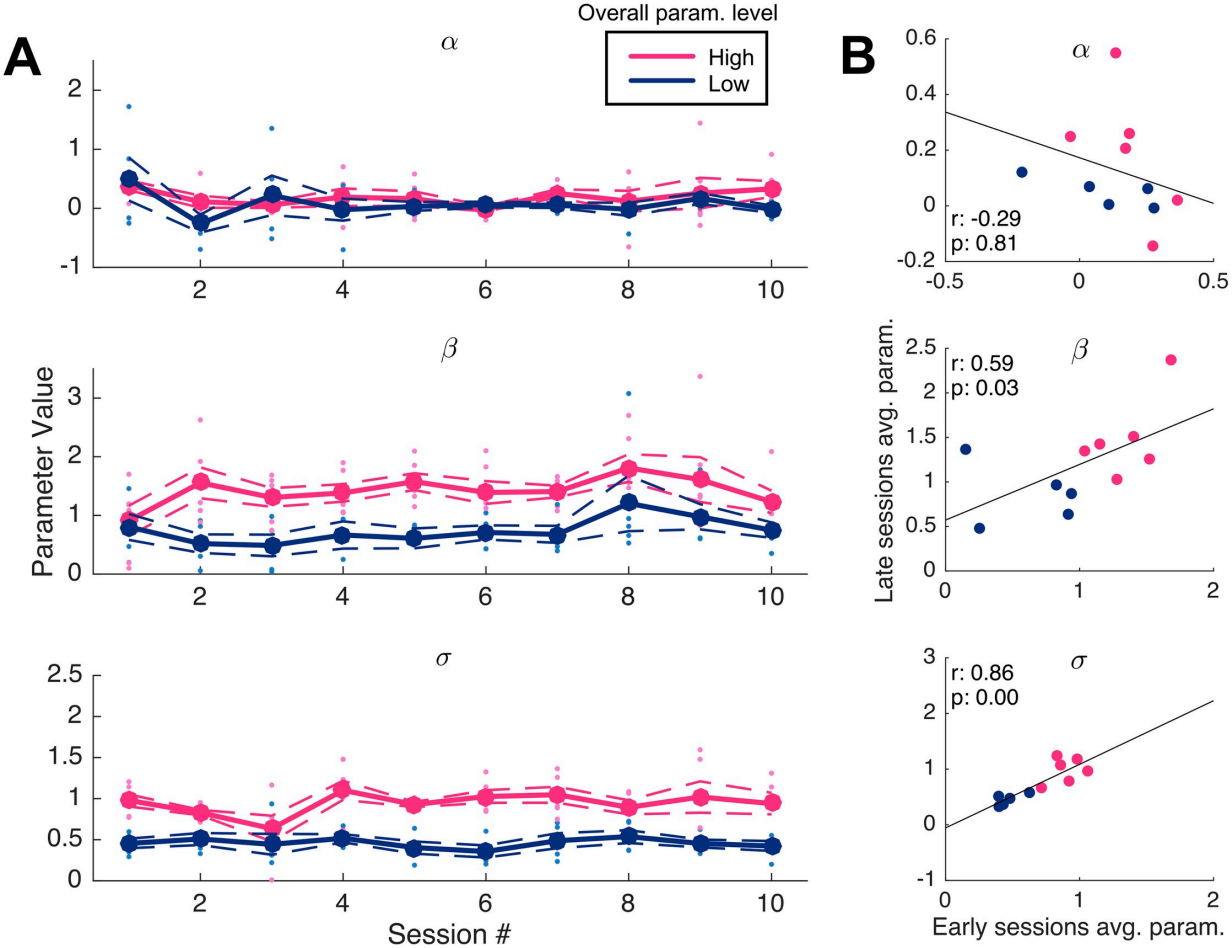
Bias parameter magnitudes across sessions. (A) Each of the three average parameter values belonging to each sub-group do not differ significantly across the 10 experiment sessions. (B) Subjects’ parameters *β* and *σ* estimated from early and late sessions (comprising the first and second halves of the study) are positively correlated.

Second, we look deeper for type stability at the subject-level across sessions. To do so, we divide the subjects into groups above and below the median for each of the model parameters, based on the subject-level parameter estimates reported in Table 3. We first confirm that the distributions of parameter estimates are not significantly different across sessions within each group. A series of one-way ANOVAs performed across the grouped parameters formally test for differences. We do not find significant differences within each half’s distribution of session-wise parameter estimates across sessions (Table 4). Moreover, the difference between sub-populations appears to be stable across sessions, as shown in Fig 7, which plots the parameter values over time for each half of the identified median split.

**Table 4:** ANOVA results on median split parameter estimates across sessions.

Median Split Type	DF	F-stat	p-value
Low *α*	F(9,50)	0.831	0.591
High *α*	F(9,40)	1.146	0.355
Low *β*	F(9,50)	1.234	0.296
High *β*	F(9,40)	0.900	0.534
Low *σ*	F(9,50)	1.464	0.187
High *σ*	F(9,40)	0.534	0.841

Finally, we ask whether mean parameter values estimated during the first half of sessions are predictive of those on the second half. The presence of positive correlations between early and late parameters (of each subject) would further support the hypothesis that bias levels remain relatively constant throughout the task. We find that this is indeed the case again for parameters *β* (*r*(8) = 0.59, *p* < 0.05) and *σ* (*r*(8) = 0.86, *p* < 0.01), the two sets of parameters that maintained the earlier differences across time from their median splits. We find no significant association between early and late parameter estimates of *α* (*r*(8) = -0.29, *p* = 0.81).

On the specific characterization of the within-subject parameter variability, S2 Appendix (Further remarks on individual distributions of bias parameters) describes several post-hoc tests on the shape of each subject’s parameter distributions. The results (Table A in S2 Appendix) suggest that bias parameters *β* estimated for each subject can be described as normally distributed with a subject-specific mean and variance. Parameter distributions and accompanying (theoretical) normal distributions are plotted for visual comparison (Fig A in S2 Appendix). Despite the inherent variability of recovered parameters from individual sessions, bias magnitudes nonetheless contain a central tendency unique to each subject.

These findings confirm a key result regarding the consistency of these biases across time. The most significant dimension of probability distortion —and also of heterogeneity across individuals—is that of repulsion versus conservatism. Individuals who are relatively high and low within these ranges maintain this relative difference throughout the course of the 10-session study. The correlation between early and late bias parameters furthermore discredits the hypothesis that there is a learning effect that diminishes these distortions across time. A set of control analyses (S3 Appendix) rule out attributes of the sampled set of true probabilities (e.g., number of switches in the underlying probabilities), as well as early experiences with the task, as determinants of these subject-specific biases. The results of these linear regression analyses are presented at the level of individual sessions and subjects within Table A in S3 Appendix.

### Response times and adjustment frequencies

To further support the claim of systematic behavioral biases, we document how the group biases correlate with other individual-specific behavioral measures within this task. Given the extensive variation on the conservatism-repulsion dimension, we test the following three post-hoc predictions: (*i*) Does variation in the distortion parameter predict the level of noise inherent in subjects’ estimates? [31] (*ii*) Are extreme responses—as in the case of repulsive biases—associated with lower response times? (*iii*) Similarly, are these extreme estimators adjusting their response sliders more frequently?

We begin with the examination of associations between bias magnitudes exhibited by each subject. We test for correlations between each subjects’ nonlinear bias parameter *β* and their respective overestimation (*α*) and randomness (*σ*) parameters. We find a significant correlation between *β* and *α* (*r*(9) = 0.60, *p* < .05); however, average *β* values do not correlate with the subjects’ associated values of *σ* (*r*(9) = 0.40, *p* = 0.11).

**Fig 8:**
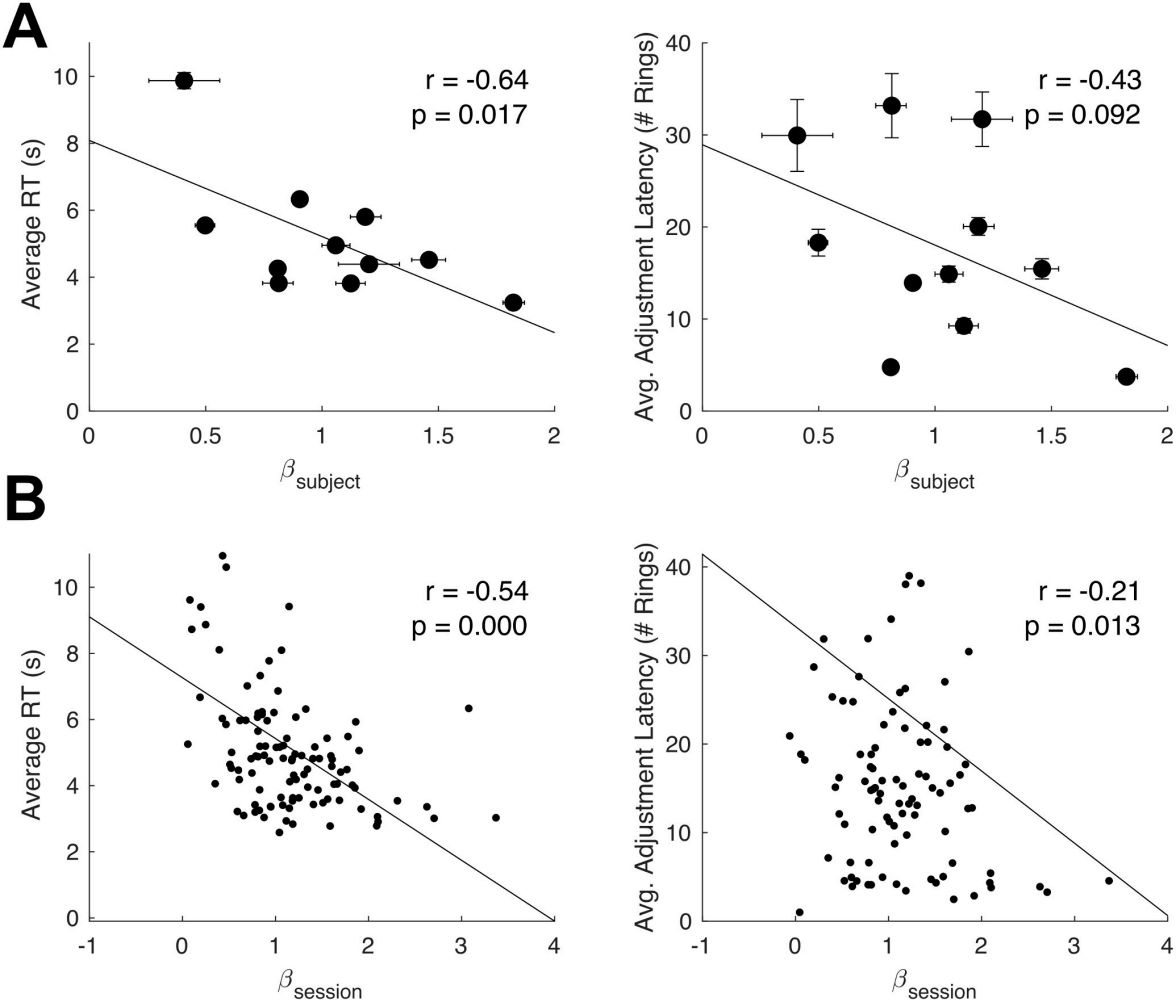
Timing-related behaviors and individual biases. (A) Increasing levels of the repulsion parameter are negatively associated with average response times (in requesting for the next ring sample). A similar negative correlation holds for the average adjustment lag (number of rings before an adjustment to the slider is made) exhibited by each subject. (B) An internal replication of both relations. Negative correlations are also observed using session-wise parameter estimates and averages.

We next confirm that individual degrees of the conservative-repulsion bias (using the M.L. estimates of *β* reported in Table 1) to be negatively and significantly correlated with average response times (*r*(9) = −0.64, *p* < .05). Similarly, the repulsion parameter values are negatively associated (at a statistically non-significant level) with the wait time between adjustments (*r*(9) = −0.43, *p* = 0.09), where the wait time is the average number of rings drawn between the subject’s revisions of estimates (Fig 8). Hence, subjects who make more conservative predictions also deliberate longer between ring draws. These subjects might also adjust their reports less frequently, waiting for more ring draws to be realized before adjusting their estimates. As an internal replication of both findings, we test for correlations between the same variables at the session level, using *β*_*session*_ values introduced earlier and the response times and adjustment latencies, now averaged within sessions. We find the same negative associations (Fig 8B) for response times (*r*(108) = −0.54, *p* < 0.001) as well as adjustment latencies (*r*(108) = −0.21, *p* = 0.01).

## Alternative forecasting models

In this section, we compare the performance of the benchmark distorted Bayesian forecast to that of three different types of forecasting models that feature prominently in the literature on probability estimation and reporting: a quasi-Bayesian model (QB), a delta rule model, and a probability theory plus noise (PTN) model. While the QB model can be seen as providing a source for how biases might arise in the benchmark model, the latter two models capture forecasting algorithms that are entirely different from the Bayesian probability theory framework. The goal of this section is to determine if any of these models provide either insight into the sources of bias estimated in the benchmark model, or a better characterization of the subjective forecasts in our data, relative to the benchmark model.

### Incorrect weighting of information as a source of bias

The distorted Bayesian forecast can be given a deeper foundation, as approximating boundedly rational probability estimation. To show this connection, we consider a quasi-Bayesian forecasting rule that has been argued to capture behavioral limitations in probability forecasting [32–34]: an incorrect weight on the likelihood ratio in the application of Bayes rule. This specification represents a specific mechanism through which subjective forecasts deviate from the Bayesian optimum, in terms of how probabilities are updated internally. This incorrect weighting of the likelihood governs how close the Quasi-Bayesian posterior remains to the prior, versus responding to new information.

To implement this model, we modify the construction of the Bayesian forecast by introducing a parameter *q* that governs the weight put on the likelihood in the updating of the posterior distribution. The Bayesian forecast features *q* = 1; a posterior distribution that is overly sensitive to new data features *q* > 1, while one that puts too much weight on the prior relative to the likelihood features *q* ∈ (0, 1). The specification also allows for incorrect use of data: estimating *q* < 0 implies that the posterior moves in the opposite direction compared with the optimal revision, given the data received.

For each participant, we estimate *q* jointly with the forecasting noise in the linear log-odds specification,

$$\begin{array}{c} log(\frac{R_{t}}{1-R_{t}})=log(\frac{Q_{t}}{1-Q_{t}})+\varepsilon_{Qt}, \end{array}$$ (4)

where *Q*_*t*_ is the quasi-Bayesian forecast and the noise is independently and identically distributed across each subject’s trials, $\varepsilon_{Qt}∼N\left(0,\sigma_{Q}^{2}\right)$.

### Experience-based forecasting alternatives

A challenge of the Bayesian and quasi-Bayesian models of probability estimation is their significant computational complexity and relatedly, their biological intractability. One possibility is that probability-encoding neurons act *as if* they were part of a system that produced distorted or quasi-Bayesian forecasts, so that even though these models may not be appropriate for describing circuit- or systems-level computations, they can nevertheless be used to understand and predict human forecasts reasonably well. Alternatively, the probability estimates we observe might be generated by an entirely different class of learning algorithms and such models might offer alternative mechanisms behind the apparent biases that resemble distortions to the Bayesian forecast.

To shed some light on how much support our data offers for each of these possibilities, we compare the Bayesian and quasi-Bayesian models to two non-Bayesian models of probability estimation: the *delta rule* and the *probability theory plus noise* (PTN) model [14]. A fundamental difference between these learning models and the models based on Bayesian probability theory is that they produce a point estimate only, rather than a posterior distribution (whether correctly calculated or distorted), and they do not use Bayes’ rule or any a priori knowledge of the ring generating process, instead relying exclusively on the experienced ring draws to form forecasts. An advantage of these models is their reduced computational burden, which makes them potential candidates for describing how the brain actually updates probabilities over the course of a task.

We first consider the basic formulation of a delta rule model, a model that is widely used in the literature, including specifically in the literature on change-point estimation [26, 35]. Error-based updating also represents a computation characteristic of midbrain dopaminergic circuits [36, 37]; incremental updating based on unexpected error signals are ubiquitous in descriptions of action values learned through reinforcement [38]. According to this model, forecasts are adjusted as a function of the discrepancy between the existing forecast and new information. Formally,

$$\begin{array}{c} D_{t}=D_{t-1}+\delta (s_{t}-D_{t-1}), \end{array}$$ (5)

where *D*_*t*_ is the forecast of the delta rule after seeing the ring realization of trial *t*, *s*_*t*_ is equal to 1 for a green ring and 0 otherwise, and *δ* is the learning parameter that governs the rate at which information decays (in this case, exponentially).

Alternatively, the PTN model employs a frequentist approach: it bases forecasts on a count of the green rings observed in a sample of ring draws a given length. This account involves the averaging of the recent past—the impact of individual past samples recently implicate episodic memory systems in the construction of learned values [39, 40]. The forecasts produced by this model also do not incorporate prior knowledge about the ring generating process. The count is subject to some probability *d* of recording each ring realization incorrectly. Let *n* denote the size of the sample of ring draws that the subject bases their forecast on, and let *d* denote the probability that a ring realization will be flipped when computing the running fraction of green rings. The expected forecast on each trial is

$$\begin{array}{c} F_{t}=\left(1-2d\right)\times \left(k_{t}/n\right)+d, \end{array}$$ (6)

where *F*_*t*_ is the PTN forecast after seeing the ring realization of trial *t*, and *k*_*t*_ is the number of green rings the participant recorded in the *n* draws ending with the draw on trial *t*.

We construct both forecasts for each participant, and, as in the Bayesian specifications, we allow for Gaussian noise in the log-odds of the forecast, to account for stochasticity at the trial level. We estimate the best-fitting parameters (*δ*, *σ*_*D*_) and (*d*, *n*, *σ*_*F*_), where the noise in each case is normally distributed with mean zero and variance $\sigma_{D}^{2}$ and $\sigma_{F}^{2}$, respectively.

### Model comparisons

Table 5 reports the estimated parameter values at the participant level. Starting with the QB models, as predicted by the theory, all the subjects estimated to have a repulsion bias (*β* > 1) also have an exponent on the likelihood function estimated above 1, and for all but one conservative subject, we estimate *q* ∈ (0, 1). Overall, the estimates of *q* are positively correlated with the non-linear bias *β* in the baseline model, despite the small number of participants.

**Table 5:** Comparison of parameter values in alternative forecasting models.

Subject	*β*	*q*	*δ*	*n*	*d*
1	1.06	1.14	0.08	13	0.08
2	0.41	0.58	0.06	3	0.31
3	0.91	0.92	0.07	14	0.08
4	1.12	1.09	0.12	11	0.10
5	0.81	0.77	0.06	11	0.13
6	1.20	1.24	0.17	4	0.10
7	0.81	1.18	0.09	16	0.11
8	1.82	1.18	0.11	4	0.10
9	0.50	0.04	0.03	23	0.23
10	1.46	1.11	0.13	6	0.06
11	1.19	1.01	0.09	15	0.04
*Corr*(⋅, *β*)	1.0	0.7	0.7	-0.4	-0.7

Note: The parameters correspond to the distorted Bayes baseline model with full parameter heterogeneity (*β*), the Quasi-Bayesian model (*q*), the delta rule (*δ*), and the Costello and Watts (2018) PTN model (*n* and *d*).

For the alternative models, we find relationships across the three types of parameters that are consistent with their respective theories (subject to the limitations of a small number of participants). As shown in Table 5, the learning rate of the delta rule is positively correlated with the non-linear bias in the benchmark model, while for the PTN model, the error rate and the sample size over which to estimate the frequency of rings are positively associated with more conservatism (namely, a smaller nonlinear bias).

**Fig 9:**
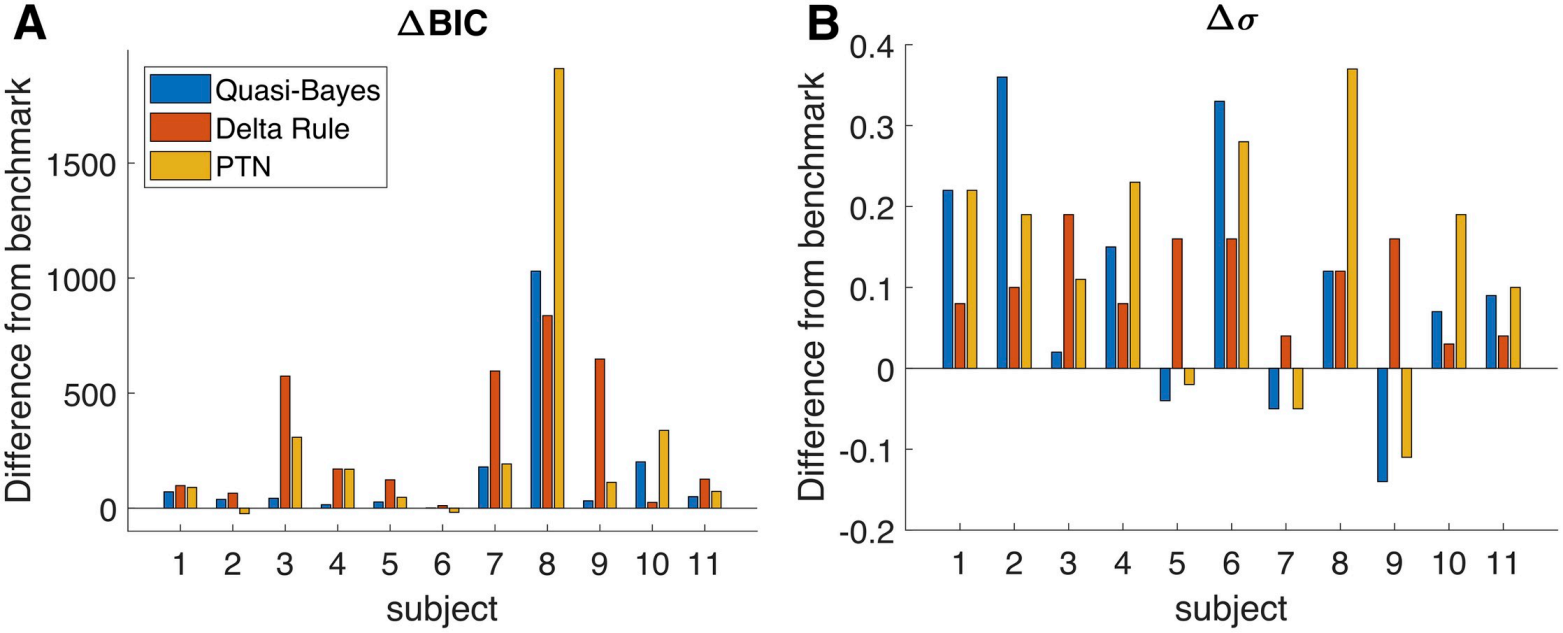
Comparisons between alternative models of probability encoding and reporting. (A) The difference in BIC values across the three different types of models, relative to the distorted Bayesian benchmark. The smallest differences are for subject 6, for whom the QB BIC is 1 point higher, the delta rule BIC is 11 points higher, and the PTN BIC is 18 points lower than the benchmark. Estimation results are based on the data conditional on adjustment for all models. The BIC levels vary across subjects depending on how frequently each subject adjusted their forecast. (B) The difference in the standard deviation of the noise associated with each model, relative to the distorted Bayesian benchmark.

Fig 9 compares the models in terms of BIC and magnitude of the noise associated with each forecast. The BIC values of the QB model are larger than those of the distorted Bayesian model for all participants, with the difference ranging from one point to over 1,000 points. The differences are modest for approximately half of the participant pool, suggesting that the QB specification has roughly comparable explanatory power for this group of participants. Nevertheless, the baseline distorted Bayesian model remains the best performing specification. Through its flexible inclusion of nonlinear biases, it appears to capture biases in probability forecasting beyond the incorrect weighting of information.

We also find no systematic evidence that either the delta rule or the PTN model outperform the distorted Bayesian benchmark: BIC values are larger for all participants for the delta rule and for all but two participants for the PTN model. The QB model is outperformed by the delta rule for two participants, and by the PTN model for two other participants, although the differences in the BIC value are quite modest. Moreover, the level of noise that each model needs to incorporate to account for the randomness in the subjective forecasts is also larger than the benchmark for all participants in the case of the delta rule and for all but three participants in the case of the PTN model. Our takeaway from this analysis is that despite their computational advantage, the experience-based models do not appear to present a compelling alternative to the distorted Bayesian benchmark model. The subjective forecasts, although nonlinearly distorted, appear to track the Bayesian estimate better than models that directly incorporate known and relevant cognitive mechanisms.

## Discussion

We find significant heterogeneity and persistent biases across subjects estimating non-stationary probabilities. Both conservative and repulsive biases are observed in different subjects, while some subjects are also approximately Bayesian (with noise). In our experimental sample, the conservative subjects roughly balance out the subjects with repulsive biases, so that median performance resembles unbiasedness. Our analyses replicate the aggregate characterization originally reported by Gallistel et al. [15], while also supporting the ubiquity of biased performance found in other experiments of probability and proportion estimation. Our results call for additional studies in order to confirm the prevalence of subject types seen here (e.g., with a large sample study). In addition, our study offers an additional instance of individual-level phenomena being occluded by analyses of pooled data [41]. Furthermore, markers of each ‘style’ of estimation appear to be stable even following extensive experience (10 sessions featuring 1,000 trials per session). We document negative correlations between the primary distortion parameter and two forms of timing behaviors: response times and delays between adjustments. Finally, fitted parameter values of error and sampling-based computational models provide a potential explanation for the directionality of observed distortions (Table 5); nonetheless, characterizations of estimates based on Bayesian forecasts offer the best explanatory power across all model types.

These individual differences may reflect variation in how much emphasis individuals place on different aspects of the computational demands of this task. In the model of Costello and Watts [14], unbiasedness occurs as a result of a balance between two different sources of bias, associated with (*i*) probability estimation in a given state and (*ii*) inference about whether the state has changed. In the case of many of our subjects, such unbiasedness is not observed at the individual level; but we might nonetheless suppose that the two sources of bias are relevant for each of our subjects, with the balance between the strength of the two biases different for different subjects. The “step-hold” pattern of slider adjustment in our experiment (as opposed to adjustment following every ring observation) can then be interpreted as resulting from subjects being engaged serially with these different cognitive tasks.

The links between individual subjects’ bias and secondary measures such as response time also suggests that differing biases may reflect differing approaches to decision making. Repulsive biases in this task appear to be associated with an inaccurate, high-frequency adjustment strategy (and vice versa with conservatism). In terms of general performance, both repulsive and conservative subjects attain lower overall payoffs compared to their unbiased peers. Nonetheless, adjustment and response speeds can be interpreted as reflecting subjects’ general approach to the task. Rather than adjusting the slider position modestly with each new piece of evidence, subjects report a new estimate only periodically. Infrequent adjustments in this paradigm might then be interpreted as reflecting implicit integration of new observations, updating internal estimates without overt adjustment [26]. In this view, subjects who produce repulsive estimates appear to have lower thresholds for updating their declared estimates of the hidden state (analogous to the definition of overconfidence by Moore and Healy [31]). The negative association between response time and repulsion further indicates that these adjustment decisions are made relatively quickly—potentially economizing on attention or cognitive resources. Thus, these individuals are more inclined to revise their current slider setting quickly and often. Interestingly, conservative subjects exhibit the longest response times, suggesting that their errors do not stem from time pressure. Rather, they may reflect an aversion to declaring extreme responses, even when responses are chosen quite deliberately. The persistence of estimation styles across sessions is also a promising indicator that these reflect *reliable* estimation techniques, as discussed in the context of other forms of probability estimation [42]. A growing body of experiments on economic decision-making has highlighted how individual differences in process measures (such as gaze time and looking preferences) correspond to apparent preferences over payoff outcomes [43–45]. In line with such findings, individual differences in this task might correspond to variation across more general psychological constructs (e.g., patience), and could also be related to differences in apparent preferences (e.g., time and risk preferences).

At present, it is difficult to distinguish between the biases in our subjects’ behavior that result from probability estimation as opposed to the detection of change points. Future experimental designs should seek to isolate one factor from the other. Indeed, in a simpler task involving the observation of short sequences of coin flips, Offerman and Sonnemans [20] find a more consistent pattern of overreaction (that they interpret as evidence of the ‘hot-hand’ effect). Conservatism is instead more consistently observed in other settings; for example, it has been associated with the influence of moderate prior expectations [46]—similar to biases that arise when incoming evidence is associated with high uncertainty [47] or low perceptual discriminability [48]. Similarly, overly extreme responses could stem from the over-weighing of recent observations [49], not unlike other general sequence effects found in perceptual judgments [50].

Another direction for future work would explore the extent to which it is possible to modify subjects’ estimation biases by changing the context in which they encounter a given decision problem. For example, experimenters might implement different payoff structures or underlying distributions of true background probabilities than those recently tested [15–17], in order to incentivize different estimation strategies. As an example, repulsive-type subjects might learn to produce conservative estimates if intermediate probabilities were indeed more likely to occur within the study. Experimental variation in visual reference points (e.g., tick marks on a response scale) has also been implicated in changing and reversing the modal bias seen in proportion estimation [8]. While we do not observe improvements in performance over time (Fig 7) within our particular environment, further experiments are necessary to deduce whether individual differences persist in different settings. In particular, the misestimation of frequencies might relate to performance in other learning paradigms. Individuals are adept at learning the payoffs associated with multiple reward streams that vary over time (e.g., [51]), and aligning rates of behavior to match associated rates of reinforcement (e.g., [52], [53]). Overall, future work should aim to causally identify the factors that promote subject-level heterogeneity in distorted estimates, e.g., by manipulating details of the experiment, or by investigating further subject demographics. The origins of such biased treatment of probabilities might then be related to other mental representations, such as those of learned reward frequencies or perceived risk.

It is intriguing that participants’ estimates are on average unbiased, despite the degree of differences in individual response patterns. In this respect, our results are in line with other observations indicating “the wisdom of the crowd” (e.g., [54], [55], [56]). It is possible that people have evolved to explore different forecasting strategies in a way that makes the group collectively approximate optimal Bayesian inference, even though individuals deviate substantially from it, as in the model of Wojtowicz [57]. Our sample is not large enough to allow us to confirm or reject any hypothesis of this kind (about how ecological contexts shape individual or population tendencies), but this would be a reasonable topic for further research.

## Supporting information

S1 Appendix Optimal Bayesian and quasi-Bayesian inference. (PDF)

S2 Appendix Further remarks on individual distributions of bias parameters. Table A. Fit results characterizing subjects’ distributions of *β* values as normally distributed. Figure A. The subject-specific distributions of parameters estimated from individual sessions.
(PDF)

S3 Appendix Effects of sampled probabilities and early task experiences. Table A. Effects of attributes of sampled true probabilities on bias parameters.
(PDF)

S1 Table Individual BIC values for the fully heterogenous model and the second-best variant for each subject. (PDF)
