# Supplementary material for: Individual differences in the perception of probability
Source: PLoS Comput Biol. 2021 Apr 1;17(4):e1008871. doi: 10.1371/journal.pcbi.1008871 (PMC8043721; doi:10.1371/journal.pcbi.1008871)
Supplement: S1 Appendix — (PDF) [file pcbi.1008871.s001.pdf]

Supporting information:

Individual differences in the perception of probability

Mel W. Khaw[1], Luminita Stevens[2], and Michael Woodford[3]

[1]Center for Cognitive Neuroscience, Duke University
[2]Department of Economics, University of Maryland
[3]Department of Economics, Columbia University

S1 Appendix. Optimal Bayesian and Quasi-Bayesian Inference

Given a sample of T observations, the Bayesian forecaster determines the posterior distribution over (n, p), where p is the most recent probability of drawing a green ring and n is the number of periods for which the current regime has lasted so far. The agent's prior is that the probability of drawing a green ring is drawn from the distribution $f(p)$ and that there is a probability δ of a new independent draw of this probability from one trial to the next.

A model of the data is specified by a probability p and a partition $\pi = \{n_i\}$ of the sample into successive regimes, where n_i is the length of regime i. Let τ_i denote the last observation of regime i. The likelihood of the most recent n observations if the regime has been p over that time is

$$L(n, p) = p^{k_n}(1-p)^{n-k_n}, \tag{1}$$

where k_n is the number of successes in the n most recent observations. Let

$$L(n) \equiv \int L(n, p) f(p) dp \tag{2}$$

and let $L_\tau(n)$ denote the average likelihood computed using the n observations ending with observation τ. The ex-ante joint probability of the model (π, p) being correct and the data being a particular observed sequence is given by

$$\mu(\pi) \prod_{i=1}^{N(\pi)-1} L_{\tau_i}(n_i) f(p) L(n, p),$$

where $N(\pi)$ is the number of regimes under partition π and μ_π is the ex-ante probability of partition π occurring in a sample of length T,

$$\mu(\pi) = (1-\delta)^{T-N(\pi)}(\delta)^{N(\pi)-1}. \tag{3}$$

Summing over the set $\Pi(n)$ of all possible partitions for which the final regime is of length n, we define

$$Q(n) \equiv \sum_{\pi \in \Pi(n)} \mu(\pi) \prod_{i=1}^{N(\pi)-1} L_{\tau_i}(n_i). \tag{4}$$

The posterior probability of (n, p) is

$$P(n, p) = \frac{Q(n) f(p) L(n, p)}{\Sigma_{n \geq 1} Q(n) L(n)}. \tag{5}$$

The expected value of p sums over all n and integrates over p using the measure $P(n,p)$. The Bayesian estimate for the probability of drawing a 1 on the next observation takes into account the fact that the regime might change on the next draw, which occurs with probability δ, and in which case, the estimate of the probability is 0.5:

$$B = (1-\delta)\int \sum_{n\geq 1} pP(n,p)dp + \frac{\delta}{2}. \tag{6}$$

To compute the quasi-Bayesian forecasts, which potentially incorrectly weight new information when updating posterior beliefs, we replace the likelihood $L(n,p)$ with $[L(n,p)]^q$, for some exponent q. The Bayesian optimum is nested under $q = 1$.

We implement the model recursively, by keeping track of $k_t(n)$, the number of green rings realized in the n observations ending with observation t, and $Q_t(n)$, the probability that the regime ending with observation t is of length n. We initialize the ring count with

$$k_t(1) = \begin{cases} 0 \text{ if red ring} \\ 1 \text{ if green ring} \end{cases}$$

and, for $1 < n \leq t$, update it recursively according to

$$k_t(n) = \begin{cases} k_{t-1}(n-1) \text{ if red ring} \\ k_{t-1}(n-1) + 1 \text{ if green ring} \end{cases}$$

$Q_t(n)$ is initialized at $Q_1(1) = 1$ and then updated recursively according to

$$Q_t(n) = \begin{cases} \delta \sum_{n=1}^{t-1} Q_{t-1}(n)L(n) \text{ for } t > 1, n = 1 \text{ [new regime]} \\ (1-\delta)\, Q_{t-1}(n) \text{ for } t > 1, 1 < n \leq t \text{ [no regime change]} \end{cases}$$

Using the values of $\{k_t(n)\}$ we compute $L(n,p)$ and $L(n)$, which, together with the values of $\{Q_t(n)\}$ yield the posterior $P(n,p)$.
