# Supplementary material for: Individual differences in the perception of probability
Source: PLoS Comput Biol. 2021 Apr 1;17(4):e1008871. doi: 10.1371/journal.pcbi.1008871 (PMC8043721; doi:10.1371/journal.pcbi.1008871)
Supplement: S2 Appendix — Table A. Fit results characterizing subjects’ distributions of β values as normally distributed. Figure A. The subject-specific distributions of parameters estimated from individual sessions. (PDF) [file pcbi.1008871.s002.pdf]

Supporting information:

Individual differences in the perception of probability

Mel W. Khaw[1], Luminita Stevens[2], and Michael Woodford[3]

[1]Center for Cognitive Neuroscience, Duke University
[2]Department of Economics, University of Maryland
[3]Department of Economics, Columbia University

S2 Appendix. Further remarks on individual distributions of bias parameters

Here we present post-hoc analyses that describe the extent to which subjects' distribution of bias parameters β can be characterized as normally distributed with individual-specific mean and variance values.

For each subject, we compute the log likelihood that each session's estimated β value was drawn from a discretized normal distribution with the means and standard deviation of their respective session parameters. We compare these likelihoods to the likelihood that subjects' parameters were drawn from a normal distribution featuring the pooled average (1.12) and standard deviation (0.63), representing the case for a non-specific pool of possible bias values.

We observe a higher relative likelihood for the subject-specific normal distribution for all subjects (ΔLL in Table A). We also present probability values from a Kolmogorov-Smirnov Goodness-of-Fit test, supporting the null hypothesis of normality in Table A (with the caveat that each test was performed with a small sample of ten observations). The empirical distribution functions for each subject are plotted in Fig A.

Table A: Fit results characterizing subjects' distributions of β values as normally distributed.

Subject	Mean	Std. Dev.	ΔLL	P-value (K-S Test)
1	1.15	0.38	2.18	0.937
2	0.76	0.99	3.77	0.225
3	0.90	0.09	15.35	0.742
4	1.39	0.49	1.68	0.699
5	0.90	0.21	7.41	0.981
6	1.29	0.43	1.77	0.506
7	0.78	0.27	6.20	0.508
8	2.03	0.63	10.73	0.474
9	0.37	0.22	13.71	0.997
10	1.46	0.49	1.22	0.436
11	1.19	0.46	1.07	0.435

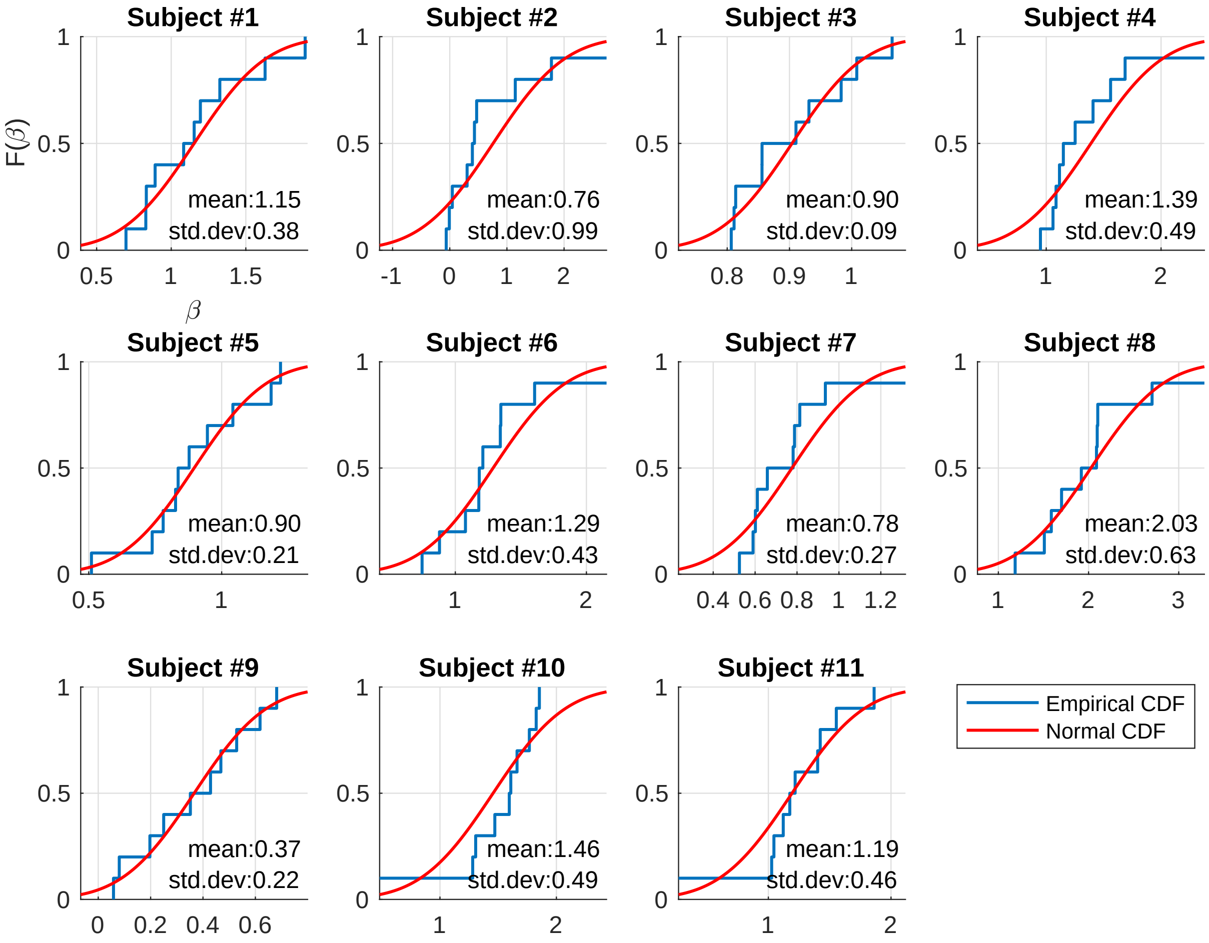

Figure A: The subject-specific distributions of β parameters estimated from individual sessions. A normal distribution's cumulative density function using the equivalent mean and standard deviation of each subject is plotted for comparison.
