# Supplementary material for: Individual differences in the perception of probability
Source: PLoS Comput Biol. 2021 Apr 1;17(4):e1008871. doi: 10.1371/journal.pcbi.1008871 (PMC8043721; doi:10.1371/journal.pcbi.1008871)
Supplement: S3 Appendix — Table A. Effects of attributes of sampled true probabilities on bias parameters. (PDF) [file pcbi.1008871.s003.pdf]

Supporting information:

Individual differences in the perception of probability

Mel W. Khaw[1], Luminita Stevens[2], and Michael Woodford[3]

[1]Center for Cognitive Neuroscience, Duke University
[2]Department of Economics, University of Maryland
[3]Department of Economics, Columbia University

S3 Appendix. Effects of sampled probabilities and early task experiences

We ran control analyses to examine whether individual differences were systematically linked to either (i) specific properties of the sampled probabilities, or (ii) early experiences with the experimental setup. To do so, we compute three attributes associated with the sampled (true) probabilities encountered by each subject: the average squared distance between true probabilities and Bayesian estimates ($Dist$), the variance of the sample of true probabilities (Var), and the number of switches in the true probabilities (Vol). $Dist$ comprises errors in estimation that arise even for an ideal observer, while Var and Vol provide measures of variability and volatility in the series of true probabilities. We first test for effects at the level of individual sessions with the following linear regression equation:

$$\beta_{session} = \theta_0 + \theta_1 Dist_{session} + \theta_2 Var_{session} + \theta_3 Vol_{session} + \epsilon \quad (1)$$

Separately, we test whether variation in the β parameters were significantly associated with the corresponding attributes of each subjects first experimental session:

$$\beta_{subject} = \theta_0 + \theta_1 Dist_{first} + \theta_2 Var_{first} + \theta_3 Vol_{first} + \epsilon \quad (2)$$

We find that none of these variables significantly accounted for the variation in β parameters found at either the across-session or across-subject level (Table A).

Table A: Effects of attributes of sampled true probabilities on bias parameters.

	Session-wise bias ($\beta_{session}$)				Individual subject bias ($\beta_{subject}$)			
	Estimate	SE	t-stat	p-value	Estimate	SE	t-stat	p-value
Distance	-13.29	28.84	-0.46	0.65	-40.84	97.26	-0.42	0.69
Variance	-0.90	1.57	-0.57	0.57	2.53	7.60	0.33	0.75
Volatility	-0.0096	0.044	-0.22	0.83	0.029	0.14	0.20	0.84
Constant	1.35	0.25	5.39	< 0.001	0.96	0.73	1.32	0.23
	Obs: 110; $RMSE$: 0.63; R^2: 0.01				Obs: 11; $RMSE$: 0.48; R^2: 0.03			
	F-stat: 0.38; p-val: 0.77				F-stat: 0.08; p-val: 0.97			
