# Supplementary material for: Individual differences in the perception of probability
Source: PLoS Comput Biol. 2021 Apr 1;17(4):e1008871. doi: 10.1371/journal.pcbi.1008871 (PMC8043721; doi:10.1371/journal.pcbi.1008871)
Supplement: S1 Table — (PDF) [file pcbi.1008871.s004.pdf]

Supporting information:
Individual differences in the perception of probability

Mel W. Khaw[1], Luminita Stevens[2], and Michael Woodford[3]

[1]Center for Cognitive Neuroscience, Duke University
[2]Department of Economics, University of Maryland
[3]Department of Economics, Columbia University

S1 Table: Individual BIC values for the fully heterogenous model and the second-best variant for each subject.

Subject	BIC	Std. Dev.	Second-best model		
			ΔBIC	Mdl. Class	Free parameters
1	1592.37	70.93	77.13	Homogenous	σ
2	794.73	15.18	33.52	Random	n/a
3	873.81	45.93	80.42	Heterogenous	α, β, σ_i
4	3249.82	80.55	16.48	Homogenous	α, σ
5	469.65	43.45	83.84	Heterogenous	α, β, σ_i
6	932.70	33.56	14.51	Homogenous	α, β, σ
7	3646.84	95.55	159.83	Heterogenous	α β_i, σ_i
8	8420.31	79.02	221.90	Heterogenous	α β_i, σ_i
9	830.89	58.71	134.12	Heterogenous	α β_i, σ_i
10	1734.70	42.83	90.92	Heterogenous	α, σ, β_i
11	1083.98	54.93	92.10	Homogenous	α, β, σ

Model classes and parameters follow the nomenclature from Table 1. Standard deviations were computed from a bootstrap procedure (5,000 iterations) – each bootstrap sample comprised an equal number of observations from the original dataset, sampled with replacement.
